# Cerebral Protection Devices during Transcatheter Interventions: Indications, Benefits, and Limitations

**DOI:** 10.1007/s11886-020-01335-9

**Published:** 2020-07-10

**Authors:** Stephan Haussig, Axel Linke, Norman Mangner

**Affiliations:** Herzzentrum Dresden, University Clinic, Department of Internal Medicine and Cardiology, Technische Universität Dresden, Fetscherstr, 76, 01307 Dresden, Germany

**Keywords:** Cerebral protection, Stroke, Cardiovascular interventions

## Abstract

**Purpose of Review:**

Stroke remains a devastating complication of cardiovascular interventions. This review is going to discuss stroke rates and outcomes in different cardiovascular procedures with a highlight on the current evidence for the use of cerebral protection devices (CPD).

**Recent Findings:**

Depending on the quality of neurological assessment, stroke occurs in up to 9.1% after TAVI, 3.9% after mitral clipping, 3.1% in LAAO patients, 0.4% after PCIs, and 1.8% after catheter ablation. CPDs are available for routine use. They are easy to use in most anatomies, feasible, and safe. Data on clinical impact and stroke reduction from RCTs are still missing.

**Summary:**

Most evidence for the routine use of CPDs exists in TAVI patients, who are at the highest risk. The PROTECTED TAVI RCT will shed more light on the clinical impact of CPD-use in TAVI patients. In other cardiovascular procedures like mitral clipping, PCIs, and ablation, the current data do not support the routine use of CPDs in these patients.

## Introduction

Stroke remains a devastating complication of cardiovascular interventions. Most of the current knowledge is derived from patients with aortic stenosis (AS) undergoing transcatheter aortic valve implantation (TAVI). However, stroke is also an apparent complication in patients undergoing mitral valve interventions, left atrial appendage occlusion (LAAO), percutaneous coronary angiography and interventions, and catheter ablation. Apart from clinically overt strokes, there are so-called silent strokes or silent brain infarcts (SBI) after cardiovascular procedures. Patients with SBIs have no signs of a clinically overt stroke, but evidence of brain injury (new cerebral embolic lesions (CEL)) mostly detected by the use of diffusion-weighted magnetic resonance imaging (DW-MRI) [1]. Recently, the Neurologic Academic Research Consortium established a consensus on the definition, classification, and assessment of neurological endpoints applicable to clinical trials of a broad range of cardiovascular interventions [2].

In this review, we aim to summarize current data on stroke rate and outcome in several transcatheter cardiovascular procedures and to highlight current evidence for the use of cerebral protection devices (CPD). Cerebral embolic protection during carotid artery stenting is beyond the scope of this review.

## Cerebrovascular Events in Cardiovascular Procedures

### TAVI

Detection of stroke and stroke rates in recent TAVI trials strongly depends on the quality of the neurological assessment. In general, stroke rates and detection of SBI are higher if the neurological assessment is performed by a neurologist or neurology fellow (e.g., 9.1% in SENTINEL US IDE control arm compared to 1.8% in the FORWARD registry) [3•, 4]. Thus, the stroke rate might be underreported in some trials. In PARTNER 3 trial, 30-day stroke rate was exceptionally low with 0.6% in the TAVI cohort [5]. This might be the result of the low-risk patient cohort; however, it is unknown whether a cerebral protection device (CPD) was used or not. Moreover, according to the trial protocol, “every effort should be made to have a neurologist (or neurology fellow) perform the NIHSS and mRS assessments.” However, if this was not possible, a certified study team member was allowed to perform the assessments [5]. Therefore, the quality of neurological assessment might have differed between both patients and centers leading to a potential reporting bias of neurological events.

Overall, a large meta-analysis of 64 studies with 72,318 TAVI patients revealed a median stroke rate of 4% (2 to 6%) [6]. In many TAVI trials, stroke rate is reported at 30 days according to the Valve Academic Research Consortium-2 (VARC-2) criteria [7]. However, a 30-day stroke rate not only reflects periprocedural strokes caused by embolization of debris but also cases of stroke due to other causes like undetected new-onset atrial fibrillation. Thus, it might be more appropriate to look at a 72-h stroke rate reflecting procedural-related strokes that might possibly be prevented by a CPD. In a patient-level propensity-matched analysis of SENTINEL US IDE, CLEAN-TAVI trial, and the SENTINEL-Ulm study, the 72-h stroke rate was 5.4% in the unprotected control group [8•].

### Mitral Valve Interventions

The stroke rate at 30 days was 0.7% in the COAPT trial [9]. Obadia and colleagues report two (1.4%) cerebral events that occurred periprocedurally and six (3.9%) ischemic strokes at 1 year in MITRA-FR [10]. In a systematic review and meta-analysis, Barros da Silva and colleagues report 61 (3.2%) pooled strokes in a total of 1881 patients after transcatheter edge-to-edge valve repair [11•]. In a small study, new CELs were detected using DW-MRI in 23 (85.7%) of 27 patients after edge-to-edge mitral valve repair [12].

Data regarding Cardioband™ (Edwards Lifesciences, Irvine, CA, USA) procedure are very limited. Nickenig and colleagues report one hemorrhagic stroke 9 days after Cardioband™ procedure performed in 31 patients [13]. One (1.6%) immediate post-procedural stroke occurred in 60 patients treated with Cardioband™ between 2013 and 2016 at eleven European institutions [14].

Overall, reported stroke rates in mitral valve interventions are considerably lower compared to TAVI. However, to date and to our best knowledge, there is no trial with mandatory neurological assessment by a neurologist at predefined time points after transcatheter mitral interventions.

### Left Atrial Appendage Occlusion

Procedure-related strokes occurred in 1.1% of the cases in the PROTECT AF trial and in 0.4% in the PREVAIL trial [15, 16]. Godino et al. report a periprocedural stroke in 6 (3.1%) of 193 left atrial appendage occlusion (LAAO) patients [17]. In the large National Cardiovascular Data Registry (NCDR) a peri-procedural ischemic stroke was apparent after 45 (0.12%) of the 38,151 LAAO procedures [18]. Only scarce data is available for new cerebral embolic lesions after LAAO. Majunke and colleagues found new CELs in 9 of 28 patients (32%) undergoing LAAO with the WATCHMAN® device (Boston Scientific, Marlborough, MA, USA) [19]. Rillig et al. found a positive correlation between the number of LAA angiographies and new CELs in 23 patients treated with the Amulet, Occlutech, or LAmbre device [20]. Laible et al. treated 21 patients with LAAO using the Amplatzer Cardiac Plug, WATCHMAN, or Amulet device. Eleven patients had a pre- and post-procedural MRI and one patient (4.8%) had a new cerebellar lesion [21].

### Percutaneous Coronary Interventions

Reported overall stroke rates in coronary interventions are low. In a pooled analysis from 11 trials including 5753 patients undergoing percutaneous coronary intervention (PCI), the 30-day stroke rate was 0.4% [22]. In another analysis of 21,510 patients who underwent cardiac catheterization and PCI at a single center over 10 years, 60 (0.28%) patients had a stroke within the first 7 days after the index procedure. Interestingly, procedural techniques changed but stroke rate remained unchanged over the years [23]. New CELs are detected in 3.3% to 34.7% of the patients undergoing PCIs [24, 25].

### Catheter Ablation

After ablation of ventricular tachycardia (VT), procedural-related strokes occur in 0.8 to 1.8% of the cases, and new CELs are detected in 58% of the patients [26–28]. The rate of procedural cerebrovascular events is lower in patients after left atrial catheter ablation (LACA) for atrial fibrillation with strokes reported in 0.1 to 0.8% of the cases, depending on the ablation method and the setting of neurological assessment [29–31]. Silent brain infarcts detected in DW-MRI are reported in up to 50% of LACA cases [32].

### Clinical Impact of CVEs

In TAVI cohorts, stroke is associated with a 6-fold increase in mortality [33]. Beside increased mortality, there are numerous adverse events associated with stroke: (1) a moderate to severe permanent disability in up to 40% of survivors, (2) a 4.7-fold increased risk for permanent work disability, (3) social isolation and significant financial strain in 80% of stroke survivors, and (4) an increased risk of readmission in patients with stroke after cardiac catheterization [23, 34–36]. The younger the patients are at the time the procedural-related stroke occurs, the higher is the long-term clinical, social, and economic impact of disability from stroke.

There is an ongoing controversial debate about the clinical impact of SBIs. However, there is growing evidence that SBIs are not benign. They are associated with an increased risk for future stroke (HR 1.5 (95%CI 1.1–2.1) to 4.7 (95%CI 2.0–11.2)) cognitive impairment and dementia [37–39]. For a detailed review on SBIs after TAVI, we recommend reading the article of Pagnesi et al. [40]

### Risk Factors for Occurrence of CVEs

Several risk factors for CVEs were identified, but they differ among studies, in particular depending on patient characteristics, including parameters, definition of events, and time points of assessment. Previous cerebrovascular disease and older age are two independent risk factors associated with early and late CVEs after TAVI and within the general population [41–45]. In an analysis of 10,982 patients from the CENTER collaboration, a GFR < 30 ml/min/1.73 cm^2^ was an independent predictor of stroke at 30 days after TAVI [44]. In another analysis, post-dilatation and valve dislodgement/embolization were independent predictors of an acute (≤ 24 h) CVE [43]. Valve in valve (VIV) procedures are discussed to have a higher risk of material embolizing and thus possibly causing a higher stroke rate. In a recent study, Eitan and colleagues compared the risk for new CEL between native valve TAVI and VIV procedures in 250 patients. Patients treated with CPDs were excluded from the analysis. A new stroke occurred in one of the 41 VIV patients (2.4%) compared to four (0.5%) of the 209 native valve TAVI patients. However, the rate of new CELs was higher in patients treated for native valve AS than VIV (153 (73.2%) vs. 21 (51.2%), *p* = 0.005) [46•].

A systematic review of 64 TAVI studies including 72,318 patients revealed female sex, chronic kidney disease, enrollment date, and new-onset atrial fibrillation as independent predictors of CVEs at 30 days after index procedure [6]. According to those risk factors derived from different studies, nearly every TAVI patients and a majority of patients undergoing other cardiovascular procedures are at high risk for stroke. Thus, it is difficult to decide which patient might benefit most from the use of a CPD, since stroke seems to be an unpredictable event. In the next chapters, we want to review different CPDs and the current evidence for the use of CPDs to prevent patients from periprocedural stroke.

## Cerebral Protection Devices

**Fig. 1:**
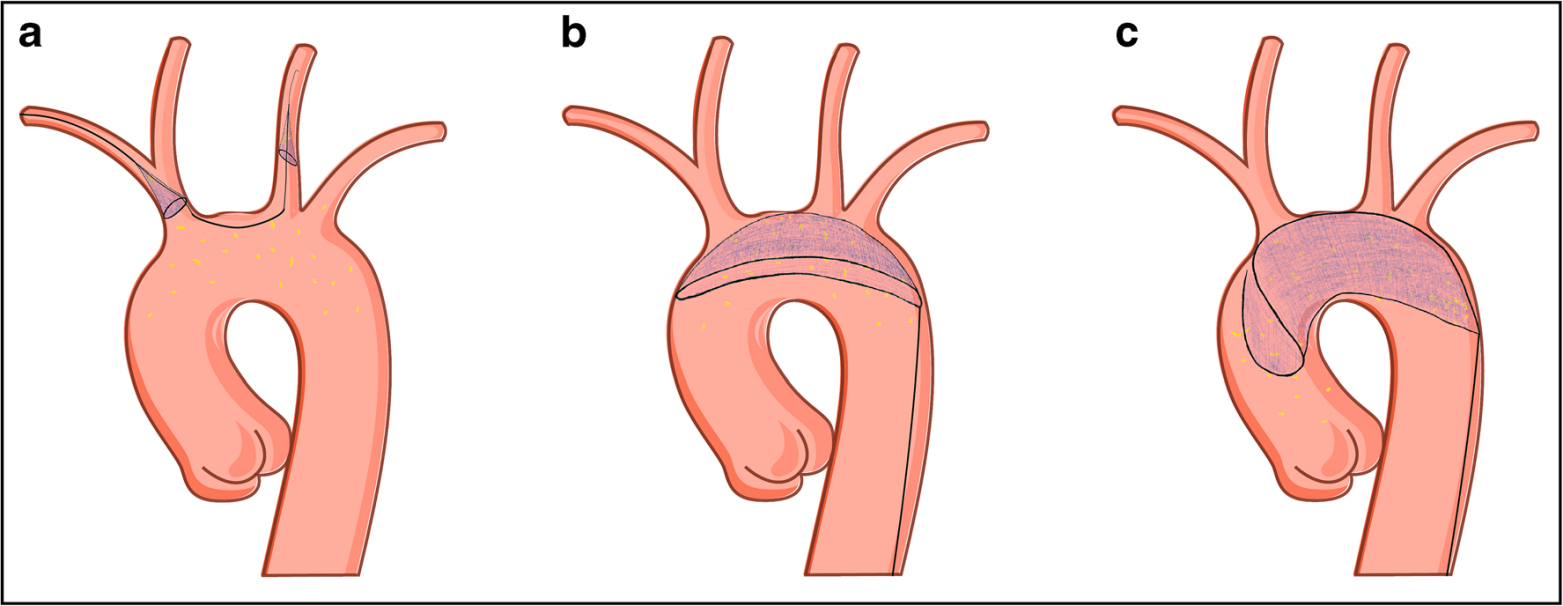
Schematic draw of a filter-based capture (**a**), deflection (**b**), and complete protection device (**c**)

Several devices have been developed to reduce cerebral embolization during TAVI (Table 1) and other cardiovascular interventions. They vary in the mechanism for protection, e.g., capture vs. deflection, but also with regard to access site and delivery sheath size. Some ideas are derived from protection devices used for cerebral embolic protection during carotid artery stenting, whose description is beyond the scope of this *review*. The most desirable characteristic of such a device is the protection of all three large branches of the aortic arch (Fig. 1), procedural stability, and a significant filter or deflection capability without causing harm to the supra-aortic vessels or the aortic arch.

**Table 1:** Cerebral protection devices

	Sentinel® cerebral protection system (Boston Scientific)	TriGuard3™ Embolic Deflection Device (Venus Medtech, Inc.)	Point-guard™ dynamic cerebral embolic protection (Transverse Medical, Inc.)	ProtEmbo® cerebral protection system (Protembis, GmbH)	Emblok™ embolic protection system (ICS, LLC.)	Emboliner™ embolic protection catheter (Emboline, Inc.)	Captis™ Embolic Protection System (Filterlex Medical Ltd.)
Mechanism	Capture	Deflection	Deflection	Deflection	Capture	Capture	Capture
Coverage	Partial	Full arch	Full arch	Full arch	Full arch	Full arch	Full arch
Access	6F, right transradial	8F, contralateral femoral	10F, contralateral femoral	6F, left transradial	12F, contralateral femoral	9F, contralateral femoral	Ipsilateral femoral
Mesh pore size	140 μm	115–145 μm	105 μm	60 μm	125 μm	150 μm	115 μm (?)
Current clinical trials	PROTECTED TAVI trial (ongoing, NCT04149535)	REFLECT trial (completed, not published)	CENTER trial (on hold)	PROTEMBO SF trial (completed, not published)	European Study Evaluating the EMBLOK EPS During TAVR (recently published, Latib et al.^55^)	SafePass 2 trial (early clinical results presented at TCT 2019	unknown
EU/USA status	CE marked/FDA approved	Investigational use	Investigational use	Investigational use	Investigational use	Investigational use	Investigational use

*F* indicates French (1F~0.33 mm); partial = two vessel protection (brachiocephalic and left common carotid artery); full arch = three vessel protection (brachiocephalic, left common carotid and left subclavian artery)

### Sentinel® Cerebral Embolic Protection System (Boston Scientific, Marlborough, MA)

The most established system is the Sentinel Cerebral Protection Device (CPD), which is a capture device consisting of two filter baskets within a single 6 F delivery catheter placed percutaneously from the right radial (preferred) or brachial artery over a 0.014-in. guide-wire. The filters are positioned in the brachiocephalic and the left common carotid arteries before the intervention and are removed afterwards. Moreover, an additional filter protecting the left vertebral artery can be used to achieve complete cerebral protection during TAVI [47]. Despite the slight increase in fluoroscopic time, the device deployment usually takes less than 10 min in 91% of cases [3•, 48].

The third generation Sentinel received the CE-mark in 2013 and the U.S. Food and Drug Administration (FDA) approved the Sentinel® CPD in 2017 for capturing and removing embolic material during TAVI procedures (class II classification), having a low-risk profile, and gaining a potential benefit in stroke prevention [49]. Postmarketing surveillance (FDA Manufacturer and User Facility Device Experience [MAUDE] database) revealed 43 reports of complications involving Sentinel® devices from 2017 through 2019. However, it is unknown how many devices were used in total, and thus, no incidence rate can be reported. Additionally, no information is available for the experience of the implanting physician with the device, and there is no data available in the MAUDE database to exclude complications caused by user error [50].

### TriGuard 3™ Embolic Deflection Device (Venus Medtech, Inc.)

The second most studied device is the TriGuard system. TriGuard 3 is the latest generation embolic deflector system from Keystone Heart (now Venus Medtech). It is designed as a deflection device covering all three supra-aortic vessels providing reduced interference with TAVI delivery systems and other heart procedure devices. It consists of a larger filter area with a smaller mesh pore size than previous generations (Table 1). TriGuard 3 includes an over the wire design via a 6 F femoral sheath for PigTail catheter placement, thereby eliminating the need for a third puncture site [51].

So far, first generations of the TriGuard have been evaluated in three prospective clinical studies of patients undergoing TAVI in the USA and Europe demonstrating numerically lower (insignificantly) stroke rates, as well as reduced total lesion volume for patients with complete coverage of all cerebral branches compared to those who were unprotected in a combined analysis [52–54]. Patients enrolled in the REFLECT trial were treated with the second and third generation TriGuard system. The study already completed enrolment, but results are not available yet.

### Point-Guard™ Dynamic Cerebral Embolic Protection (Transverse Medical, Inc.)

The Point-Guard system is a deflection device providing coverage of all great arch vessels and is aimed to protect the patient from embolic debris during TAVI or other left-sided procedures. The device is designed as a flexible nitinol frame with filter mesh wrapped around its perimeter and a supporting extension at its distal end. Its isolation zone is thought to dynamically stabilize the device during positioning and reducing device migration and decoupling. No clinical data have been published so far, and currently, the Point-Guard is only for investigational use [51].

### Emblok™ Embolic Protection System (Innovative Cardiovascular Solutions, LLC)

Emblok EPS is thought to protect the cerebrum as well as abdominal and peripheral vasculature during TAVI or other left-sided heart procedures. The system is designed as an embolic filter with an incorporated PigTail catheter that can be advanced simultaneously through a single femoral puncture site. It consists of a 125-μm pore-sized filter system, which is positioned in the aorta and is expected to accommodate in anatomies up to 35 mm in diameter due to its flexible nitinol design [51].

In a first in man study of 20 TAVI patients, the Emblok system was successfully positioned in every patient. There were no procedural-related strokes at 30 days. Debris was captured in 18 (90%) patients. However, new CELs were detected in 95% of the patients. The authors conclude that the use of the Emblok system is feasible and safe [55•]. Larger studies are necessary to confirm the results and to further evaluate the clinical benefit.

### ProtEmbo® Cerebral Protection System (Protembis GmbH)

This device is intended to protect all three supra-aortic vessels by deflecting embolic debris. The low-profile design is made for delivery by left radial access; thereby, avoiding interference with the TAVI delivery systems and manipulating the carotid arteries. The heparin-coated mesh has the smallest pore size (60 μm) among all available CEPs. For this reason, it might even safeguard the cerebrum from smaller sized debris [51]. The PROTEMBO SF Trial (NCT 03325283) is set to demonstrate the safety and feasibility of the ProtEmbo® System when used to provide embolic protection during TAVI.

### Other CEP Devices/Novel Technologies Providing Full-Body Embolic Protection

Not only embolism to the brain has disastrous consequences but also to peripheral arteries and organs, in particular, renal failure due to embolism is a major concern [56]. Filterlex Medical Ltd. recently introduced the Captis™ Embolic Protection System—a system consisting of a filter-covered collapsible frame and filter pockets that aims for “full-body embolic protection” by deflecting embolic particles away from the aortic arch and capturing them in the pockets to avoid renal embolism. The Emboliner™ Embolic Protection Catheter (Emboline, Inc.) is another CEP system aiming for complete cerebral and peripheral protection [51]. It is being evaluated in the SafePass 2 Trial with results presented at TCT 2019, showing the feasibility of the device. No peer-reviewed publication is available so far.

## Cerebral Protection in Various Settings

### TAVI

The greatest experience for the use of CPDs exists in TAVI patients. The Sentinel CPD is the most-studied device in the field of cerebral protection during TAVI. It has been assessed in several investigations starting with first-in-man studies followed by two randomized controlled trials (CLEAN-TAVI and MISTRAL-C) which applied second and third-generation Sentinel devices [48, 57–60]. The latter ones proved a significant reduction in number and volume of new cerebral lesions in protected areas by DW-MRI scans after TAVI. The stroke rates, however, were not statistically different between patients randomized to the Sentinel CPD and those in the control group, although trials were underpowered for clinical outcomes [48, 60]. Afterwards, the Sentinel CPD was tested in a multicenter, single-blinded RCT—the SENTINEL US IDE study, in which 363 patients undergoing TAVI were randomized to cerebral protection or no protection, with further randomization to MRI and neurocognitive examination or safety follow-up [3•]. The device was associated with a favorable safety profile, and in almost every case, embolic debris was captured. The 30-day stroke rate, although statistically not significant, was reduced by 39% (control, 9.1% vs. intervention, 5.6%; *P* = 0.25). Reduction in new lesion volume on MRI was numerically lower, but not statistically different. In a post hoc analysis adjusting for valve type and baseline lesion volume, there was a considerable reduction in new lesion volume in protected cerebral territories with the Sentinel CPD. Furthermore, the efficacy was attenuated in patients receiving the Sapien S3 valve. This correlation could be the consequence of generally lower rates of embolization with this device and, therefore, the treatment effect is more difficult to establish. Consequently, another multicenter trial currently enrolling participants (PROTECT TAVI) is set to re-evaluate the cerebral protection on MRI findings in a four-armed study (balloon- vs. self-expandable valves) (NCT 02895737).

None of the aforementioned studies was able to prove a significant reduction in clinical stroke rate, in particular, due to a lack of statistical power and the remaining question about the appropriate primary endpoint. A CPD provides protection only during the procedure and, therefore, stroke rate at 30 days does not seem to be a proper endpoint. A patient-level pooled analysis including 1306 patients from CLEAN-TAVI, SENTINEL US IDE, and the SENTINEL-Ulm study showed a significant 65% relative risk reduction of strokes occurring up to 72-h post-procedure [8•]. This endpoint seems to be plausible for a CPD since more than 50% of major strokes occur within the first 2 days after TAVI and are considered as periprocedural strokes [43, 61]. However, this analysis also has its limitations extensively discussed in the manuscript and an accompanying Editorial preventing us to definitively answer the question whether the SENTINEL CPD prevents clinical stroke or not [8, 62]. Therefore, the multicenter RCT PROTECTED TAVI (Stroke Protection with Sentinel During Transcatheter Aortic Valve Replacement) was designed to assess the impact of the SENTINEL CPD with the primary endpoint rate of stroke through 72-h post-TAVI procedure or discharge (whichever comes first) (NCT04149535). We hope that this trial will close the gap of knowledge in cerebral protection during TAVI.

However, some considerations on the device design of the SENTINEL CPD should be kept in mind while interpreting existing data and waiting for new ones. As mentioned above, the current generation Sentinel CPD provides filter protection to three of the four major arteries to the cerebrum, leaving the left vertebral artery unprotected. In general, the left vertebral artery is more dominant than the right vertebral artery and therefore has a larger vascular territory [63]. An additional filter for the left vertebral artery could be deployed in nine out of eleven cases in a small feasibility study. It contained debris in an equal amount and size as the Sentinel filters itself indicating that the left vertebral artery is an important entry route for debris to the brain during TAVI [47]. Besides the partial protection issue, there is currently only one size of filter available, which might prevent complete sealing in certain anatomies. In the future, next-generation embolic protection devices, as shown above, are developed to demonstrate different strategies to address these challenges.

### Mitral Valve Interventions

There is no RCT available comparing mitral valve intervention with and without the use of a CPD, so that the only evidence exists from case reports and the initial experience of two centers using CPDs during mitral clipping. Frerker et al. successfully used the Sentinel CPDs in 14 patients undergoing MitraClip implantation. Debris was found in the filters of all patients. Histopathological analysis revealed acute thrombus and foreign material as the most common tissue types. No procedural-related CVE occurred in the 14 patients, and no DW-MRI was performed. The authors conclude that the use of a CPD in a MitraClip setting was feasible and safe [64]. As debris was captured in every case and thus patients were prevented from embolization, CVEs were potentially prevented. However, according to the scarce data available, routine use of CPDs during mitral interventions cannot be recommended.

### Left Atrial Appendage Occlusion

Published experience with CPDs in LAAO is limited to some cases. In a study of 28 patients with evidence of left atrial thrombus treated with LAAO, CPDs were used in six (21.4%) cases (1 Sentinel, 4 TriGuard, 1 FilterWire EZ). Macroscopically visible debris was captured in one case. No histological analyses and no MRIs were performed. No procedural-related complications occurred, neither in patients with CPD nor in those without CPD [65]. Overall, a systematic review of all published cases of LAAO in patients with LAA thrombus identified 17 patients treated with cerebral protection. CPDs used included Triguard, Sentinel, and FilterWires. All procedures were performed without any procedural complications [66]. There are no larger studies or even RCTs available for the use of CPDs during LAAO. However, in a setting with evidence of left atrial thrombus, CPDs might potentially prevent patients from brain damages due to debris embolization.

### Percutaneous coronary interventions

We performed an extensive literature search of all publications in the PubMed and Medline databases and found no study with the above-mentioned devices used in a setting of coronary diagnostics or interventions. Due to the low event rate in these procedures, it seems infeasible to conduct an RCT that is adequately powered.

### Catheter Ablation

Evidence for the use of CPD during ablation is limited to case reports and the analysis of 11 patients undergoing ventricular tachycardia (VT) ablation. In these patients, the Sentinel® system was successfully deployed and retrieved in all cases. Again, debris was captured in all cases. Histopathological analysis revealed acute thrombus, tissue from the arterial wall, and foreign material as the most common types of tissue captured by the filter system. No CVEs related to the procedure occurred. No DW-MRI was performed. The authors conclude that the use of a CPS was feasible and safe [67].

## Conclusion

Stroke complicating cardiovascular procedures remains a major health issue, especially in TAVI patients. Risk factors predicting the occurrence of CVEs after cardiovascular procedures vary. Given the potential risk of a CVE in every patient, most evidence for the routine use of a CPD exists in TAVI patients, who are at the highest risk of experiencing a procedural-related stroke. As indications for TAVI expand to lower-risk patients, it is of utmost importance to make the procedure as safe as possible. The PROTECTED TAVI (Stroke Protection with Sentinel During Transcatheter Aortic Valve Replacement) RCT (NCT04149535) will shed more light on the clinical impact of CPD-use in an all-comers TAVI cohort. In other cardiovascular procedures like mitral clipping, PCI, and ablation, the incidence of CVEs is low, and the current data do not support the routine use of CPDs in these patients.

## Abbreviations

AS: Aortic stenosis
CEL: Cerebral embolic lesion
CVE: Cerebrovascular event
CPD: Cerebral protection device
DW-MRI: Diffusion-weighted magnetic resonance imaging
GFR: Glomerular filtration rate
LAAO: Left atrial appendage occlusion
PCI: Percutaneous coronary intervention
SBI: Silent brain infarct
TAVI: Transcatheter aortic valve implantation
VIV: Valve in valve
VT: Ventricular tachycardia
